# Adjuvant chemotherapy for locally advanced upper tract urothelial carcinoma: updated results of the Seoul National university hospital experience

**DOI:** 10.1590/S1677-5538.IBJU.2015.0009

**Published:** 2015

**Authors:** Hyung Suk Kim, Joong Sub Lee, Chang Wook Jeong, Cheol Kwak, Hyeon Hoe Kim, Ja Hyeon Ku

**Affiliations:** 1 Department of Urology, Seoul National University College of Medicine, Seoul, Korea

**Keywords:** Urinary Tract, Carcinoma, Transitional Cell, Chemotherapy, Adjuvant, Survival

## Abstract

**Objectives:**

The objective of this study was to update the long-term outcome in the treatment of locally advanced upper tract urothelial carcinoma (UTUC) after radical nephroureterectomy (RNU) regarding the role of adjuvant chemotherapy.

**Materials and methods:**

Clinical data from 138 patients who underwent RNU for locally advanced UTUC (pT3/4 or pN+) were analyzed.

**Results:**

The adjuvant chemotherapy group comprised 66 patients, and other 72 patients did not receive adjuvant chemotherapy. Cisplatin-based chemotherapy was the most common regimen, depending on the patient's eligibility and renal function. The median follow-up period was 48.7 months (interquartile range: 29.2-96.9 months). The 3-and 5-year disease-specific survival (DSS) rates were 76.0% and 69.9% for the non-adjuvant chemotherapy group versus 74.6% and 54.5% for the adjuvant chemotherapy group (p=0.301, log-rank test). Overall survival (OS) rates for the same time period were 70.1% and 62.9% for the non-adjuvant chemotherapy group versus 73.8% and 53.2% for the adjuvant chemotherapy group (p=0.931, log-rank test). On multivariate analysis, adjuvant chemotherapy could not predict DSS and OS after surgery. When patients who received cisplatin-based adjuvant chemotherapy (n=59) were compared to those who did not receive adjuvant chemotherapy, similar results were found.

**Conclusions:**

There does not appear to be a significant DSS or OS benefit associated with adjuvant chemotherapy. Prospective randomized clinical trials are necessary to verify the effect of adjuvant chemotherapy on locally advanced UTUC.

## INTRODUCTION

Upper tract urothelial carcinoma (UTUC) is a rare disease that accounts for approximately 5% of all urothelial malignancies (1). Although radical nephroureterectomy (RNU) has been considered standard care for treating localized UTUC, 45-60% of patients with locally advanced disease will relapse after extirpative surgery alone (2). In a large multicenter collaborative study of 1.363 patients treated with RNU, Margulis et Al. (3) reported 5-year survival rates of 74.7%, 54%, 35.3%, and 12.2% for pT2, pT3, N+and pT4, respectively. Contemporary analyses indicate that there has been no improvement in survival rates in the past several decades for patients with high-grade disease (4).

Adjuvant chemotherapy with agents for metastatic disease may be reasonable in treating locally advanced UTUC associated with poor survival. However, there is no standardized therapy conferring a survival benefit after RNU, as there have been no controlled trials that explored the efficacy of adjuvant chemotherapy in this setting. Most evidence for the treatment of patients with UTUC may be extrapolated from experience with bladder cancer.

The rarity of UTUC has resulted in a paucity of literature on adjuvant chemotherapy and its role in the treatment of high-risk UTUC (5). Previously, we reported the efficacy of adjuvant chemotherapy in patients with invasive UTUC (6). In this study, we sought to give an update by reporting the long-term outcome and role of adjuvant chemotherapy in the treatment of locally advanced UTUC after RNU.

## MATERIALS AND METHODS

**Figure 1:**
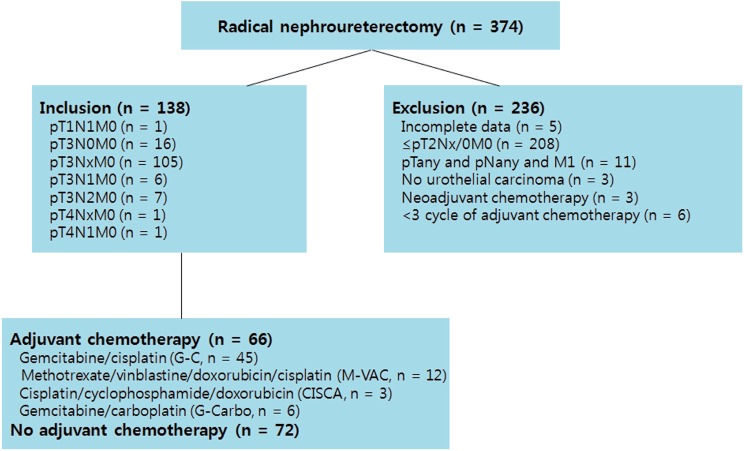
Study flow diagram.

This study was approved by the institutional review board. We performed a retrospective review of 374 patients who underwent radical nephroureterectomy (RNU) at Seoul National University Hospital from 1993 to 2010. RNU was performed according to standard procedures, and the regional lymph nodes were generally resected if intraoperatively palpable or preoperatively enlarged during evaluation. Patients with incomplete data, localized disease (≤ pT2Nx/0M0), distant metastasis (pTany and pNany and M1), no urothelial carcinoma, administration of neoadjuvant chemotherapy or administration of less than 3 cycles of adjuvant chemotherapy were excluded. To meet criteria for adjuvant chemotherapy, treatment must have been started within 3 months of undergoing RNU. Cisplatin-based chemotherapy was the most common regimen, depending on patient eligibility and renal function, as described previously (Figure-1) (6).

Pathological specimens were evaluated by a staff pathologist with genitourinary expertise. All specimens were histologically confirmed to be urothelial carcinoma. Staging was done according to the 2010 American Joint Committee on Cancer classification and grading according to the 1998 WHO system. Lymphovascular invasion (LVI) was defined as the presence of tumor cells within an endothelium-lined space without underlying muscular walls. The presence of concomitant carcinoma in situ (CIS) was assessed in every representative section. Tumor location was defined as renal pelvic, ureteral or both. Tumor multifocality was defined as the synchronous presence of 2 or more pathologically confirmed tumors in any location (renal pelvis, ureter or both). Tumor necrosis was defined as the presence of microscopic coagulative necrosis in more than 10% of the tumor.

Patients were evaluated every 3-4 months for the first two years, every 6 months for the next two years, and then annually thereafter. Follow-up consisted of history taking, physical examination, blood tests, urine cytology, cystoscopy, chest X-ray, abdominopelvic computed tomographic (CT) scan, and bone scan. Survival was evaluated from the date of surgery to last follow-up or death. Patients who were alive with or without disease were censored from the relevant analyses. Cause of death was determined by the responsible physicians and death certificates. Perioperative deaths occurring within 30 days of surgery were censored from disease-specific survival analyses.

### Statistical Analyses

For statistical analysis, the characteristics of adjuvant and non-adjuvant groups were compared. A chi-squared or Fisher's exact test was used for categorical variables and a Student's t-test for age and body mass index. Outcomes were measured by disease-specific survival (DSS) and overall survival (OS) based on chemotherapy status between the cohorts. Survival was calculated using the Kaplan-Meier method and compared using a log-rank test. To adjust for the effect of potential confounders, multivariate analyses using Cox proportional hazards model were conducted. Significant variables showing less than 0.05 of two sided p-value in the univariate analyses were entered into a multivariate analysis. The assessed variables were gender, age, body mass index, American Society of Anesthesiologists (ASA) score, previous or concomitant bladder cancer, preoperative ureteroscopy, bladder cuffing, tumor location, multifocality, hydronephrosis, tumor grade, concomitant CIS, LVI, tumor necrosis, margin status and pN stage. Pathologic T stage was not included in the multivariate model, as most pathologic T stage was pT3. However, administration of adjuvant chemotherapy was regarded as a main variable of interest and forced into the multivariate model. The same analysis was also performed for testing in a subgroup that received only cisplatin-based adjuvant chemotherapy. All statistical tests were performed with SPSS software (SPSS Inc., Chicago, IL, USA). All reported P values were two-sided, and significance was set at P<0.05.

## RESULTS

Table-1 compares the characteristics of patients who did not receive adjuvant chemotherapy (n=72) and those who received adjuvant chemotherapy (n=66) or cisplatin-based adjuvant chemotherapy (n=59). Patients who received adjuvant chemotherapy were younger than those who did not (p=0.001). The adjuvant chemotherapy group was more likely to have a lower ASA score (p=0.006), less previous or concomitant bladder cancer (p=0.001) and more concomitant CIS (p=0.014). Other variables were similar between groups.

**Table 1:** Patient characteristics.

Variables		No ACH	ACH	P value†
**Total**		72	66	
**Gender**				**0.276**
	Male	58 (80.6%)	48 (72.7%)	
	Female	14 (19.4%)	18 (27.3%)	
Age, year		67.3 (57.4-73.0)	60.3 (54.1-65.7)	**0.001**
BMI, cm/kg^2^		24.4 (22.4-25.6)	23.9 (21.2-25.7)	**0.552**
**ASA score**				**0.006**
	1	21 (29.2%)	30 (45.5%)	
	2	41 (56.9%)	35 (53.0%)	
	3	10 (13.9%)	1 (1.5%)	
**Bladder cancer** *				**0.001**
	No	51 (70.8%)	61 (92.4%)	
	Yes	21 (29.2%)	5 (7.6%)	
**Preoperative ureteroscopy**				**0.650**
	No	62 (86.1%)	55 (83.3%)	
	Yes	10 (13.9%)	11 (16.7%)	
**Bladder cuffing**				**0.311**
	No	17 (23.6%)	11 (16.7%)	
	Yes	55 (76.4%)	55 (83.3%)	
**Tumor location**				**0.219**
	Renal pelvis	41 (56.9%)	29 (43.9%)	
	Ureter	19 (26.4%)	24 (36.4%)	
Both		12 (16.7%)	13 (19.7%)	
**Multifocality**				**0.956**
	Absent	57 (79.2%)	52 (78.8%)	
	Present	15 (20.8%)	14 (21.2%)	
**Hydronephrosis**				**0.522**
	Absent	41 (56.9%)	34 (51.5%)	
	Present	31 (43.1%)	32 (48.5%)	
**Pathologic T stage**				**0.062**
	pT1	2 (2.8%)	0 (0.0%)	
	pT3	70 (97.2%)	64 (97.0%)	
pT4		0 (0.0%)	2 (3.0%)	
**Tumor grade**				**0.073**
	G1	6 (8.3%)	1 (1.5%)	
	G2	39 (54.2%)	33 (50.0%)	
	G3	27 (37.5%)	32 (48.5%)	
**Concomitant CIS**				**0.014**
	Absent	71 (98.6%)	58 (87.9%)	
	Present	1 (1.4%)	8 (12.1%)	
**LVI**				**0.280**
	Absent	56 (77.8%)	46 (69.7%)	
	Present	16 (22.2%)	20 (30.3%)	
**Necrosis**				**0.473**
	Absent	65 (90.3%)	57 (86.4%)	
	Present	7 (9.7%)	9 (13.6%)	
**Margin status**				**0.479**
	Negative	69 (95.8%)	61 (92.4%)	
	Positive	3 (4.2%)	5 (7.6%)	
**Pathologic N stage**				**0.080**
	pN0	9 (12.5%)	8 (12.1%)	
	pNx	60 (83.3%)	46 (69.7%)	
	pN+	3 (4.2%)	12 (18.2%)	

Data presented are number (%) or median (interquartile range).

† Compared to no adjuvant chemotherapy group.

* Previous or concomitant.

**ACH:** adjuvant chemotherapy; **BMI:** body mass index; **ASA:** American Society of Anesthesiologists; **CIS:** carcinoma in situ; **LVI:** lymphovascular invasion.

**Figure 2:**
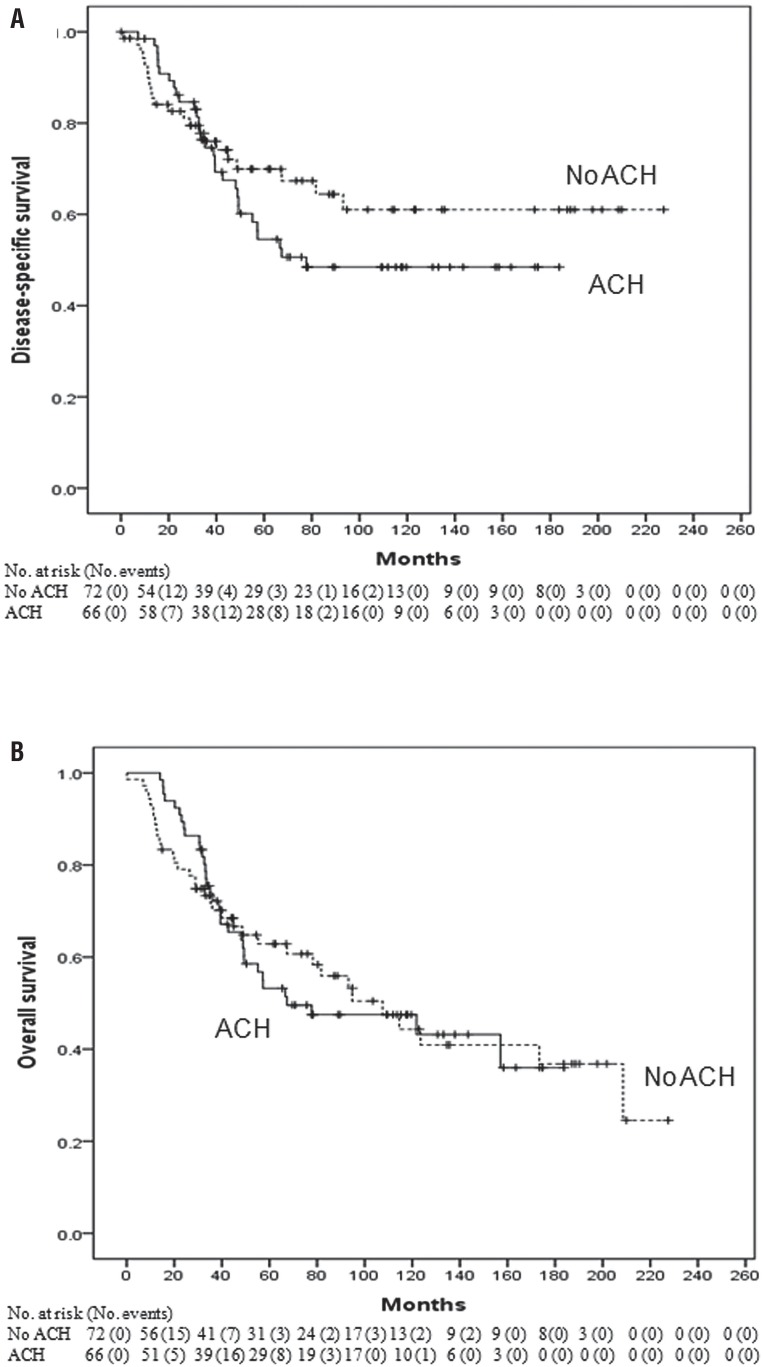
Kaplan-Meier analysis for (A) disease-specific survival and (B) overall survival after radical nephroureterectomy stratified by the administration of adjuvant chemotherapy in all patients.

Overall median follow-up period was 48.7 months (interquartile range: 29.2-96.9 months). The median follow-up was 46.8 months (interquartile range: 28.9-101.3 months) for the non-adjuvant chemotherapy group and 52.8 months (interquartile range: 33.3-110.1 months) for the adjuvant chemotherapy group (p=0.469). In the entire population, 69 (50.0%) died of any cause and 52 deaths (37.7%) were attributable to UTUC. The 3-and 5-year DSS rates were 76.0% and 69.9% for the non-adjuvant chemotherapy group versus 74.6% and 54.5% for the adjuvant chemotherapy group (p=0.301, log-rank test) (Figure-2A). OS rates for the same time period were 70.1% and 62.9% for the non-adjuvant chemotherapy group versus 73.8% and 53.2% for the adjuvant chemotherapy group (p=0.931, log-rank test) (Figure-2B).

Table-2 shows the multivariate Cox regression model for predicting DSS after surgery in total patients. Bladder cuffing, LVI, and margin status were significantly associated with DSS, while other variables were not. In multivariate analysis, bladder cuffing was the sole independent prognostic factor for DSS (p=0.002). Univariate analysis revealed that age, ASA score, previous or concomitant bladder cancer, bladder cuffing, LVI and margin status were significant predictors of OS. In the multivariate model, age (p=0.005), previous or concomitant bladder cancer (p=0.031), bladder cuffing (p=0.005) and margin status (p=0.027) remained significant predictors of OS. In contrast, ASA score and LVI were not predictors of OS. Adjuvant chemotherapy could not predict DSS and OS after surgery.

**Table 2:** Multivariate Cox proportional hazards regression analysis of disease-specific survival and overall survival.

		DSS		OS	
		HR (95% CI)	P value	HR (95% CI)	P value
Age				1.045 (1.013-1.077)	0.005
**AsA score**					
	1			Reference	
	2/3			1.327 (0.723-2.435)	0.362
**Bladder cancer** *					
	No			Reference	
	Yes			1.905 (1.061-3.422)	0.031
**Bladder cuffing**					
	Yes	Reference		Reference	
	No	2.615 (1.430-4.782)	0.002	2.211 (1.266-3.862)	0.005
**LvI**					
	Absent	Reference		Reference	
	Present	1.791 (0.994-3.226)	0.052	1.669 (0.947-2.944)	0.077
**Margin status**					
	Negative	Reference		Reference	
	Positive	2.458 (0.938-6.443)	0.067	1.879 (1.074-3.286)	0.027
**ACH**					
	No	Reference		Reference	
	Yes	1.255 (0.705-2.237)	0.440	1.430 (0.809-2.526)	0.218

* Previous or concomitant.

DSS, disease-specific survival; OS, overall survival; HR, hazard ratio; CI, confidence interval; ASA, American Society of Anesthesiologists; LVI, lymphovascular invasion; ACH, adjuvant chemotherapy.

**Supplementary Figure-1:**
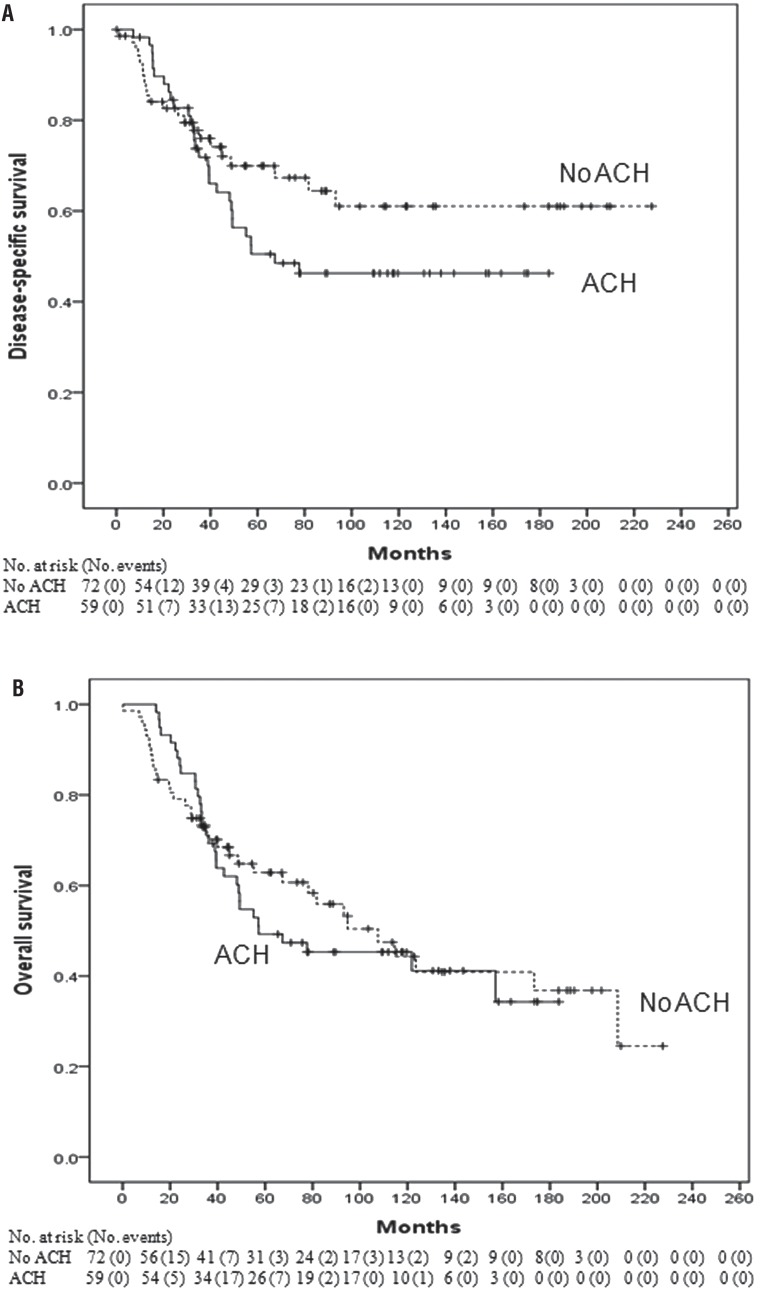
Kaplan–Meier analysis for (A) disease-specific survival and (B) overall survival after radical nephroureterectomy stratified by the administration of adjuvant chemotherapy in patients who received cisplatin-based adjuvant chemotherapy or not.

We compared the non-adjuvant chemotherapy group (n=72) to the cisplatin-based adjuvant chemotherapy group (n=59) (Supplementary Table-1). There was no significant difference between the two groups in terms of DSS (p=0.193, log-rank test) (Supplementary Figure-1A) and OS (p=0.719, log-rank test) (Supplementary Figure-1B). Supplementary Table-2 shows the multivariate Cox regression model for predicting DSS and OS after surgery. In univariate analysis, bladder cuffing, LVI, and margin status were significantly associated with DSS. Multivariate analysis revealed that bladder cuffing (p=0.001) and LVI (p=0.045) were independent prognostic factors for DSS. Univariate analysis showed a statistically significant association between OS and age, ASA score, previous or concomitant bladder cancer, bladder cuffing, LVI and margin status. In multivariate analysis, age (p=0.002), previous or concomitant bladder cancer (p=0.034), bladder cuffing (p=0.002) and LVI (p=0.029) were independent prognostic factors for OS. Adjuvant chemotherapy could not predict DSS and OS after surgery.

**Supplementary Table-1:** Patient characteristics.

Variables		No ACH	ACH (cisplatin-based only)	P value†
Total		72	59	
**Gender**				**0.298**
	Male	58 (80.6%)	43 (72.9%)	
	Female	14 (19.4%)	16 (27.1%)	
Age, year		67.3 (57.4-73.0)	60.3 (54.2-65.7)	0.001
BMI, cm/kg^2^		24.4 (22.4-25.6)	23.8 (21.2-25.7)	0.476
**ASA score**				**0.017**
	1	21 (29.2%)	25 (42.4%)	
	2	41 (56.9%)	33 (55.9%)	
	3	10 (13.9%)	1 (1.7%)	
**Bladder cancer** *				**0.003**
No		51 (70.8%)	54 (91.5%)	
Yes		21 (29.2%)	5 (8.5%)	
**Preoperative ureteroscopy**				**0.628**
No	62 (86.1%)	49 (83.1%)	
Yes	10 (13.9%)	10 (16.9%)	
**Bladder cuffing**				**0.348**
	No	17 (23.6%)	10 (16.9%)	
	Yes	55 (76.4%)	49 (83.1%)	
**Tumor location**				**0.138**
	Renal pelvis	41 (56.9%)	24 (40.7%)	
	Ureter	19 (26.4%)	23 (39.0%)	
	Both	12 (16.7%)	12 (20.3%)	
**Multifocality**				**0.868**
	Absent	57 (79.2%)	46 (78.0%)	
	Present	15 (20.8%)	13 (22.0%)	
**Hydronephrosis**				**0.486**
	Absent	41 (56.9%)	30 (50.8%)	
	Present	31 (43.1%)	29 (49.2%)	
**Pathologic T stage**				**0.066**
	pT1	2 (2.8%)	0 (0.0%)	
	pT3	70 (97.2%)	57 (96.6%)	
	pT4	0 (0.0%)	2 (3.4%)	
**Tumor grade**				**0.106**
	G1	6 (8.3%)	1 (1.7%)	
	G2	39 (54.2%)	30 (50.8%)	
	G3	27 (37.5%)	28 (47.5%)	
**Concomitant CIS**				**0.022**
	Absent	71 (98.6%)	52 (88.1%)	
	Present	1 (1.4%)	7 (11.9%)	
**LVI**				0.387
	Absent	56 (77.8%)	42 (71.2%)	
	Present	16 (22.2%)	17 (28.8%)	
**Necrosis**				**0.336**
	Absent	65 (90.3%)	50 (84.7%)	
	Present	7 (9.7%)	9 (15.3%)	
**Margin status**				**0.466**
	Negative	69 (95.8%)	54 (91.5%)	
	Positive	3 (4.2%)	5 (8.5%)	
**Pathologic N stage**				**0.028**
	pN0	9 (12.5%)	6 (10.2%)	
	pNx	60 (83.3%)	41 (69.5%)	
	pN+	3 (4.2%)	12 (20.3%)	

Data presented are number (%) or median (interquartile range).

† Compared to no adjuvant chemotherapy group.

* Previous or concomitant.

ACH, adjuvant chemotherapy; BMI, body mass index; ASA, American Society of Anesthesiologists; CIS, carcinoma in situ; LVI: lymphovascular invasion.

**Supplementary Table-2:** Multivariate Cox proportional hazards regression analysis of disease-specific survival and overall survival in patients who received cisplatin-based adjuvant chemotherapy or not.

		DSS		OS	
		HR (95% CI)	P value	HR (95% CI)	P value
Age				1.050 (1.018-1.084)	0.002
**AsA score**					
	1			Reference	
	2/3			1.310 (0.706-2.431)	0.392
**Bladder cancer** *					
	No			Reference	
	Yes			1.880 (1.048-3.372)	0.034
**Bladder cuffing**					
	Yes	Reference		Reference	
	No	2.708 (1.473-4.978)	0.001	2.399 (1.374-4.189)	0.002
**LVI**					
	Absent	Reference		Reference	
	Present	1.834 (1.014-3.317)	0.045	1.868 (1.066-3.273)	0.029
**Margin status**					
	Negative	Reference		Reference	
	Positive	2.261 (0.860-5.948)	0.098	1.852 (0.761-4.512)	0.175
**ACH**					
	No	Reference		Reference	
	Yes	1.374 (0.766-2.464)	0.287	1.647 (0.911-2.977)	0.098

* Previous or concomitant.

DSS, disease-specific survival; OS, overall survival; HR, hazard ratio; CI, confidence interval; ASA, American Society of Anesthesiologists; LVI, lymphovascular invasion; ACH, adjuvant chemotherapy.

## DISCUSSION

Although UTUC is morphologically similar to bladder cancer, there are occasional phenotypic and genotypic (genetic and epigenetic) differences between UTUC and bladder cancer (4). Furthermore, the natural history of UTUC is different from bladder cancer, with >60% of UTUCs and only 15-25% of bladder cancer presenting with invasion at diagnosis (1, 7). Therefore, data generated from bladder cancer studies cannot always be extrapolated to patients with UTUC (8).

Urothelial carcinoma of the bladder is considered to be relatively chemosensitive. The survival benefit of neoadjuvant chemotherapy before radical cystectomy in patients with invasive bladder cancer has been demonstrated (9). However, the efficacy of adjuvant chemotherapy after radical cystectomy remains controversial (10). Results of adjuvant chemotherapy studies for UTUC are also contradictory. For UTUC, adjuvant chemotherapy provided a therapeutic benefit in some studies (6, 11, 12), while there was no significant difference in outcomes between adjuvant and non-adjuvant chemotherapy groups in others (13–18). This discordance probably accounts for the inconsistent use of chemotherapy for locally advanced UTUC. Still, most patients are not candidates for cisplatin-based chemotherapy after RNU, primarily due to impaired renal function. Lane et al. (19) reported that 61% of all patients and 49% of high-risk patients who could have received chemotherapy preoperatively were unable to receive treatment after RNU.

There is no definite evidence showing that conventional M-VAC regimen significantly prolongs survival as an adjuvant treatment arm for patients with locally advanced UTUC. Lee et al. (18) investigated 27 patients who underwent RNU for pT3N0M0 UTUC with a median follow-up of 47 months. Sixteen patients received chemotherapy (M-VAC regimen with three-four cycles) after RNU, and 11 did not. No evidence of significant differences in recurrence-free survival and DSS could be found between the two groups. In the study by Soga et al. (17), adjuvant M-VAC could prevent bladder tumors following surgery for UTUC but did not show a survival benefit.

Hellenthal et al. (16) published data from an international multicenter study of 542 patients with pT3 or higher UTUC. Of these, 121 patients received adjuvant chemotherapy and chemotherapy that was cisplatin-based in 89% of cases (59% M-VAC and 20% GC). No significant differences in DSS or OS between the two groups were found. The selection of patients with more advanced disease could account for this finding. Another multi-institutional study analyzed 627 patients with T3 or higher UTUC, with 140 patients receiving adjuvant chemotherapy (mostly platinum-based). There was no evidence of extension of DSS or OS for patients treated with adjuvant chemotherapy (14). In other studies, chemotherapy was only administered to patients with higher grade and stage tumors, including metastatic disease. Thus, it is likely that patient selection contributed to inconsistent findings. A multi-center study from 10 Canadian academic centers revealed that adjuvant chemotherapy was not prognostic for improved DSS or OS (15).

However, retrospective studies have identified a benefit from adjuvant chemotherapy. Suzuki et al. (11) investigated the effectiveness of adjuvant chemotherapy in 56 patients with locally advanced bladder cancer or UTUC. Twenty patients underwent adjuvant chemotherapy (M-VAC or MEC) and 36 patients were controls. Adjuvant chemotherapy had a positive survival benefit in patients with node-positive disease, but did not affect survival of all patients. There was no distinction between UTUC and bladder cancer, making it difficult to draw definite conclusions for UTUC. Kawashima et al. (12) evaluated the data of 93 patients with pT3N0M0, and 38 received platinum-based adjuvant chemotherapy. In multivariate analysis, adjuvant chemotherapy was significantly associated with DSS.

Meta-analysis of adjuvant chemotherapy for UTUC demonstrated DSS and OS benefit with cisplatin-based adjuvant chemotherapy (20). However, most studies examining adjuvant chemotherapy were retrospective and may suffer from substantial selection biases. First, patients with the worst prognostic factors were selected to receive adjuvant chemotherapy compared to counterparts undergoing observation; the proportion of patients who had pN+ disease and received adjuvant chemotherapy was higher than those not receiving adjuvant chemotherapy (15, 16). Second, there are few studies with more than 50 patients, due to the low frequency of UTUC. Finally, the utility of adjuvant chemotherapy in UTUC may be limited given the decline in renal function following RNU, and renal excretion and inherent nephrotoxicity of cisplatin. The proportion of patients receiving adjuvant chemotherapy was three to four times smaller than those receiving surgery alone. Patients receiving adjuvant chemotherapy may have better renal function and performance status. Despite possible benefits, there is insufficient evidence to recommend routine use of cisplatin-based adjuvant chemotherapy for UTUC (20).

Previously, we reported on 43 patients with a tumor stage pT2 or higher without metastasis (6). Twenty-two patients received adjuvant chemotherapy. All regimens contained platinum with the M-VAC scheme used most often. The follow-up period totaled 30.7 months. Results showed higher DSS and OS rates for patients with adjuvant chemotherapy. However, the study included a small number of patients and may have a selection bias if the chemotherapy group had a better performance status or less comorbidity than the non-chemotherapy group.

In this update, which is the largest single-center study to date to the best of our knowledge, we enrolled 138 patients with pT3, pT4, or N+and M0 UTUC. Sixty-six patients underwent adjuvant chemotherapy, and 72 patients were solely controlled. The median follow-up period was 48.7 months. We found that adjuvant chemotherapy did not significantly correlate with DSS or OS in patients with high-risk disease compared to patients receiving no adjuvant treatment. This study included a homogeneous group of patients with stage III or IV UTUC who initially received the same surgical treatment at a single institution.

Despite this advantage, limitations include the small number of patients and its retrospective non-randomized nature. Furthermore, the regimen and number of chemotherapy cycles varied. Still, we didn't consider disease-free survival (DFS) as a potential endpoint other than OS and DSS. If we analyzed the DFS in UTUC patients receiving adjuvant chemotherapy, it might have been that adjuvant chemotherapy prolongs the DFS. A large prospective randomized trial to verify our findings is expected, though it will be difficult to perform due to low incidence of UTUC.

## CONCLUSIONS

There does not appear to be a significant DSS or OS benefit associated with adjuvant chemotherapy. A prospective randomized clinical trial is needed to verify the effect of adjuvant chemotherapy on locally advanced UTUC and to determine whether this is due to the inherent biases of retrospective analysis, the limited efficacy of adjuvant chemotherapy, or the use of suboptimal regimen. In addition, efforts should be made to develop new chemotherapeutic agents and establish reliable criteria for patient selection in performing adjuvant chemotherapy.

## Abbreviations

ASA: = American Society of Anesthesiologists
CIS: = carcinoma in situ
DSS: = disease-specific survival
GC: = gemcitabine and cisplatin
LVI: = Lymphovascular invasion
MEC: = methotrexate, epirubicin, and cisplatin
M-VAC: = methotrexate, vinblastine, adriamycin and cisplatin
OS: = overall survival
RNU: = radical nephroureterectomy
UTUC: = upper tract urothelial carcinoma

## References

[1] Rouprêt M, Babjuk M, Compérat E, Zigeuner R, Sylvester R, Burger M (2013). European guidelines on upper tract urothelial carcinomas: 2013 update. Eur Urol.

[2] Ozsahin M, Zouhair A, Villà S, Storme G, Chauvet B, Taussky D (1999). Prognostic factors in urothelial renal pelvis and ureter tumours: a multicentre Rare Cancer Network study. Eur J Cancer.

[3] Margulis V, Shariat SF, Matin SF, Kamat AM, Zigeuner R, Kikuchi E (2009). Outcomes of radical nephroureterectomy: a series from the Upper Tract Urothelial Carcinoma Collaboration. Cancer.

[4] Brown GA, Busby JE, Wood CG, Pisters LL, Dinney CP, Swanson DA (2006). Nephroureterectomy for treating upper urinary tract transitional cell carcinoma: Time to change the treatment paradigm?. BJU Int.

[5] Leow JJ, Martin-Doyle W, Fay AP, Choueiri TK, Chang SL, Bellmunt J (2014). A systematic review and meta-analysis of adjuvant and neoadjuvant chemotherapy for upper tract urothelial carcinoma. Eur Urol.

[6] Kwak C, Lee SE, Jeong IG, Ku JH (2006). Adjuvant systemic chemotherapy in the treatment of patients with invasive transitional cell carcinoma of the upper urinary tract. Urology.

[7] Babjuk M, Burger M, Zigeuner R, Shariat SF, van Rhijn BW, Compérat E (2013t). EAU guidelines on non-muscle-invasive urothelial carcinoma of the bladder: update 2013. Eur Urol.

[8] van Oers JM, Zwarthoff EC, Rehman I, Azzouzi AR, Cussenot O, Meuth M (2009). FGFR3 mutations indicate better survival in invasive upper urinary tract and bladder tumours. Eur Urol.

[9] Winquist E, Kirchner TS, Segal R, Chin J, Lukka H, Genitourinary Cancer Disease Site Group (2004). Neoadjuvant chemotherapy for transitional cell carcinoma of the bladder: a systematic review and meta-analysis. J Urol.

[10] Advanced Bladder Cancer (ABC) Meta-analysis Collaboration (2005). Adjuvant chemotherapy in invasive bladder cancer: a systematic review and meta-analysis of individual patient data Advanced Bladder Cancer (ABC) Meta-analysis Collaboration. Eur Urol.

[11] Suzuki S, Shinohara N, Harabayashi T, Sato S, Abe T, Koyanagi T (2004). Impact of adjuvant systemic chemotherapy on postoperative survival in patients with high-risk urothelial cancer. Int J Urol.

[12] Kawashima A, Nakai Y, Nakayama M, Ujike T, Tanigawa G, Ono Y, Kamoto A (2012). The result of adjuvant chemotherapy for localized pT3 upper urinary tract carcinoma in a multi-institutional study. World J Urol.

[13] Kim TS, Oh JH, Rhew HY (2013). The efficacy of adjuvant chemotherapy for locally advanced upper tract urothelial cell carcinoma. J Cancer.

[14] Vassilakopoulou M, de la Motte Rouge T, Colin P, Ouzzane A, Khayat D, Dimopoulos MA, Papadimitriou CA (2011). Outcomes after adjuvant chemotherapy in the treatment of high-risk urothelial carcinoma of the upper urinary tract (UUT-UC): results from a large multicenter collaborative study. Cancer.

[15] Yafi FA, Tanguay S, Rendon R, Jacobsen N, Fairey A, Izawa J (2014). Adjuvant chemotherapy for upper-tract urothelial carcinoma treated with nephroureterectomy: assessment of adequate renal function and influence on outcome. Urol Oncol.

[16] Hellenthal NJ, Shariat SF, Margulis V, Karakiewicz PI, Roscigno M, Bolenz C (2009). Adjuvant chemotherapy for high risk upper tract urothelial carcinoma: results from the Upper Tract Urothelial Carcinoma Collaboration. J Urol.

[17] Soga N, Arima K, Sugimura Y (2008). Adjuvant methotrexate, vinblastine, adriamycin, and cisplatin chemotherapy has potential to prevent recurrence of bladder tumors after surgical removal of upper urinary tract transitional cell carcinoma. Int J Urol.

[18] Lee SE, Byun SS, Park YH, Chang IH, Kim YJ, Hong SK (2006). Adjuvant chemotherapy in the management of pT3N0M0 transitional cell carcinoma of the upper urinary tract. Urol Int.

[19] Lane BR, Smith AK, Larson BT, Gong MC, Campbell SC, Raghavan D (2010). Chronic kidney disease after nephroureterectomy for upper tract urothelial carcinoma and implications for the administration of perioperative chemotherapy. Cancer.

[20] Leow JJ, Martin-Doyle W, Fay AP, Choueiri TK, Chang SL, Bellmunt J (2014). A systematic review and meta-analysis of adjuvant and neoadjuvant chemotherapy for upper tract urothelial carcinoma. Eur Urol.

